# Supplementary figures and images for: The metabolic ability of swallowtails results in the production of bioactive substances from plant components
Source: PLoS One. 2025 Jul 23;20(7):e0321438. doi: 10.1371/journal.pone.0321438 (PMC12286346; doi:10.1371/journal.pone.0321438)

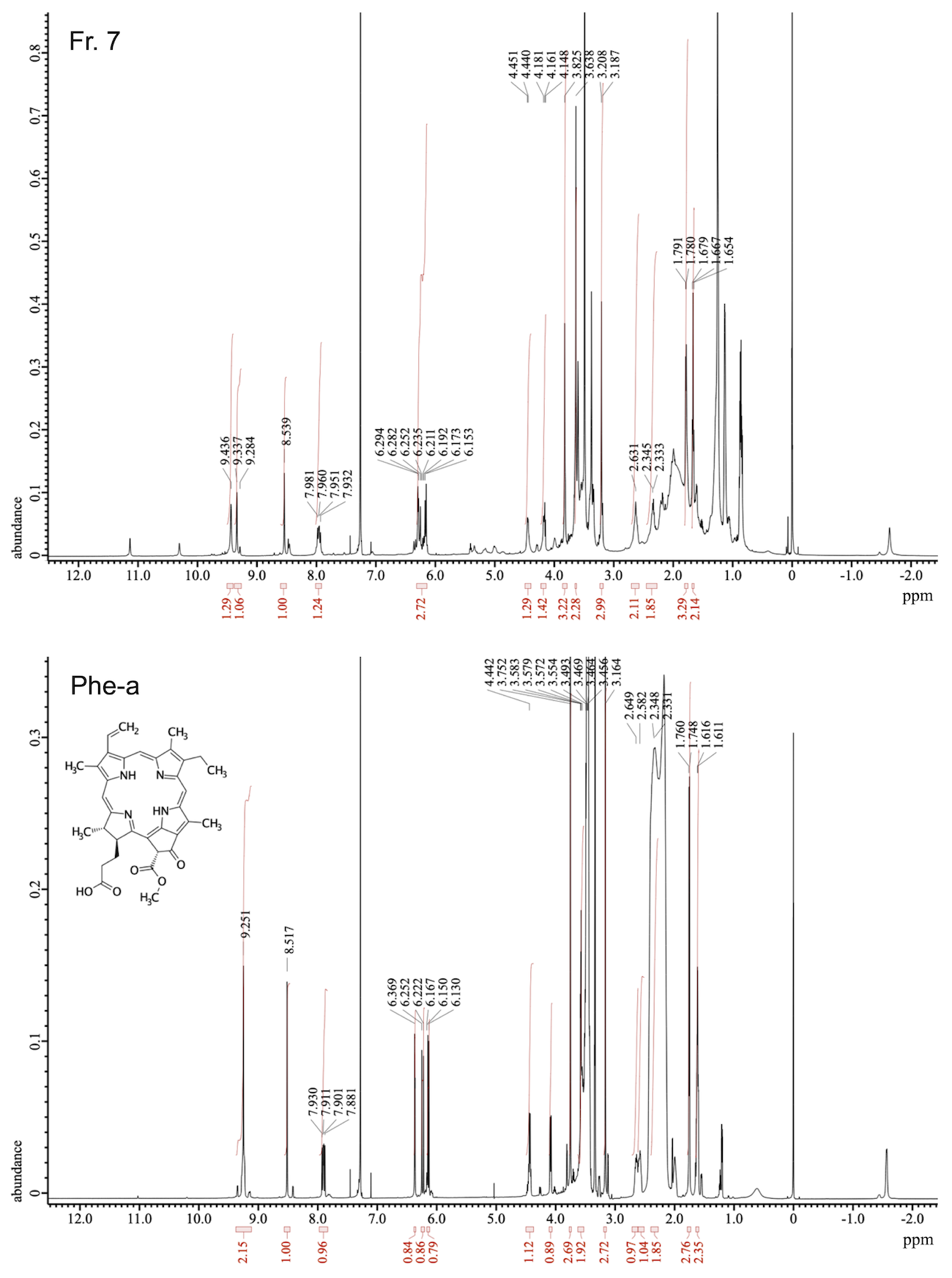

Supplement: S1 Fig — (TIFF) [file pone.0321438.s001.tiff]

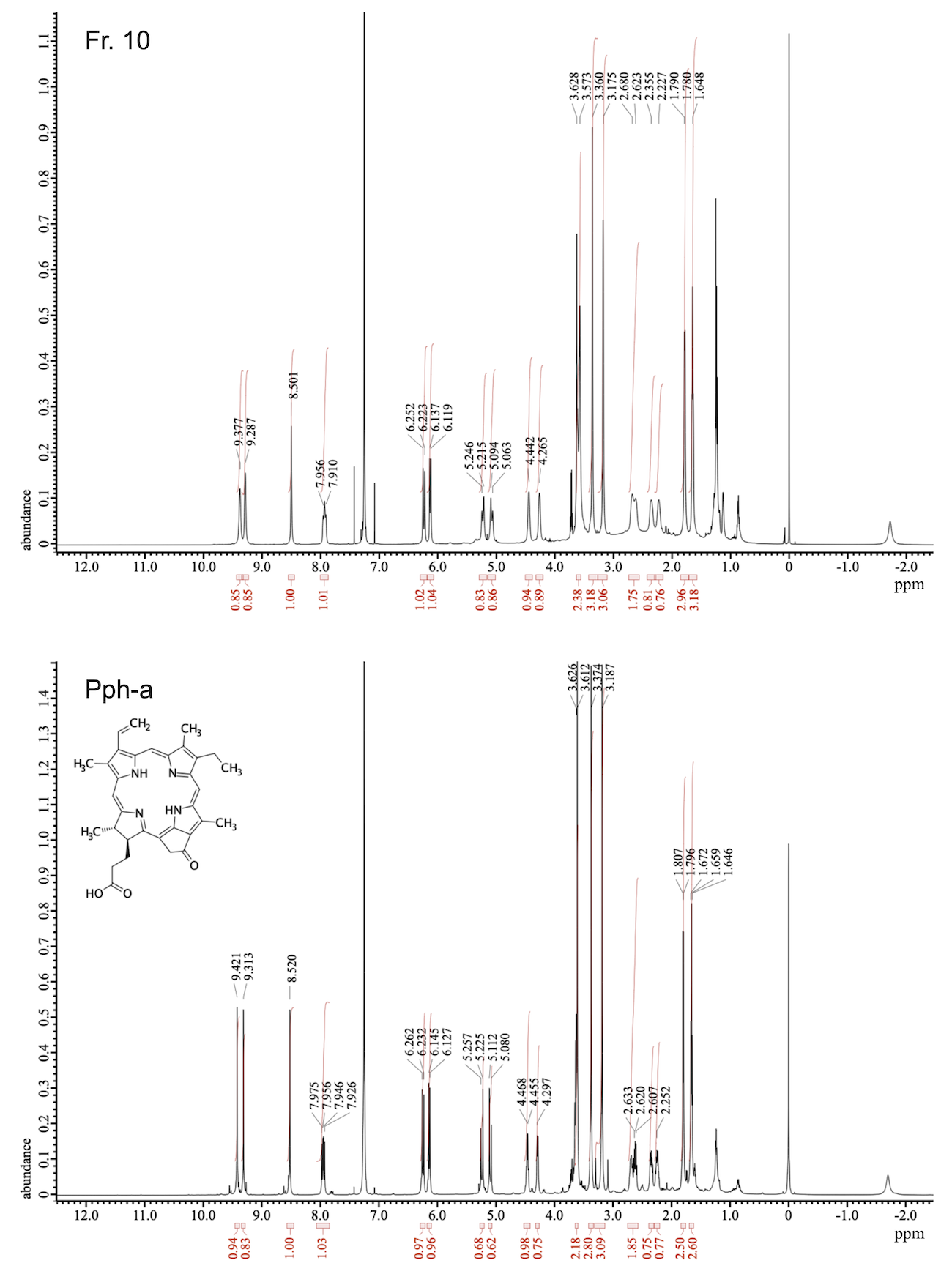

Supplement: S2 Fig — (TIFF) [file pone.0321438.s002.tiff]

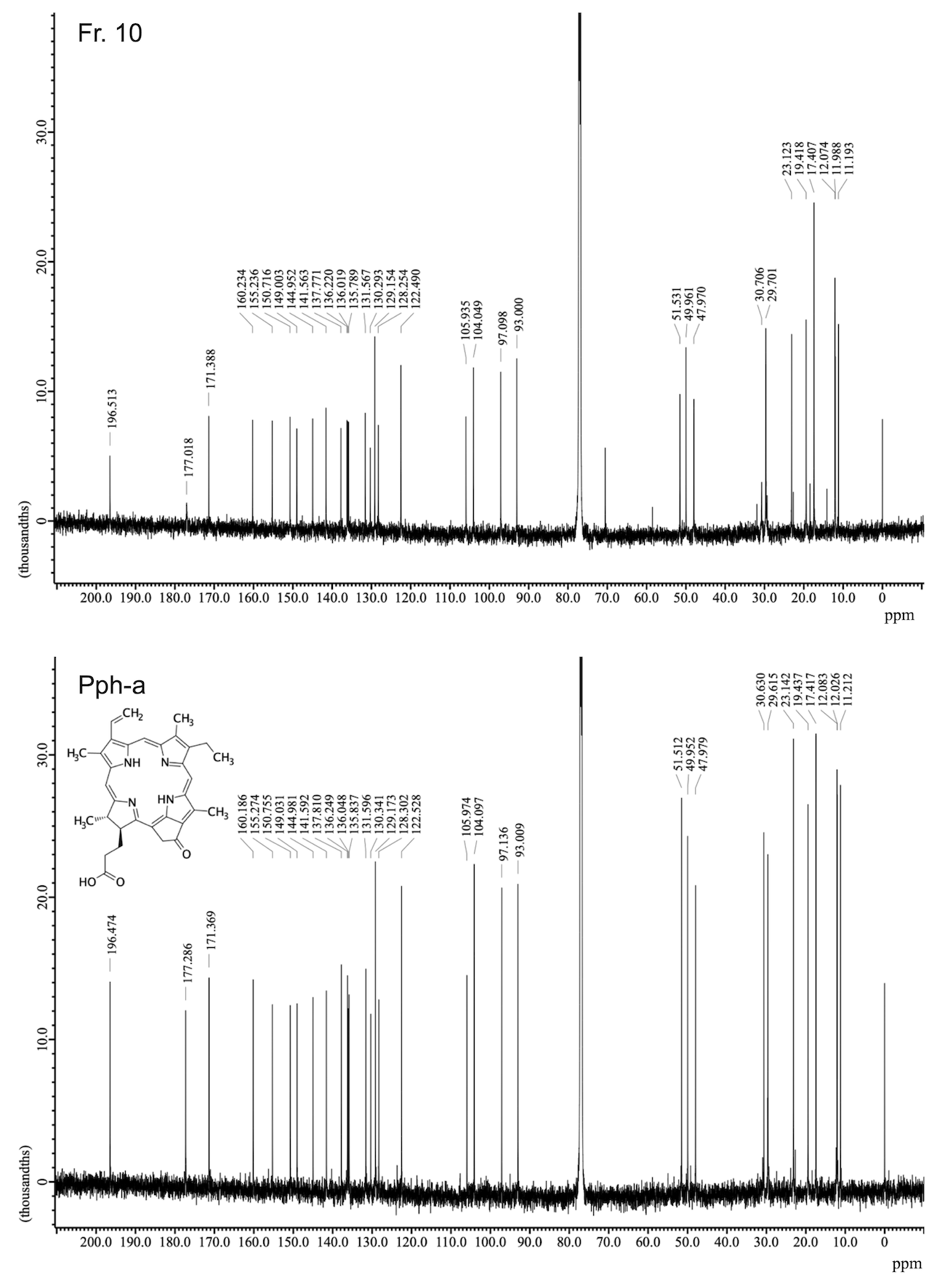

Supplement: S3 Fig — (TIFF) [file pone.0321438.s003.tiff]

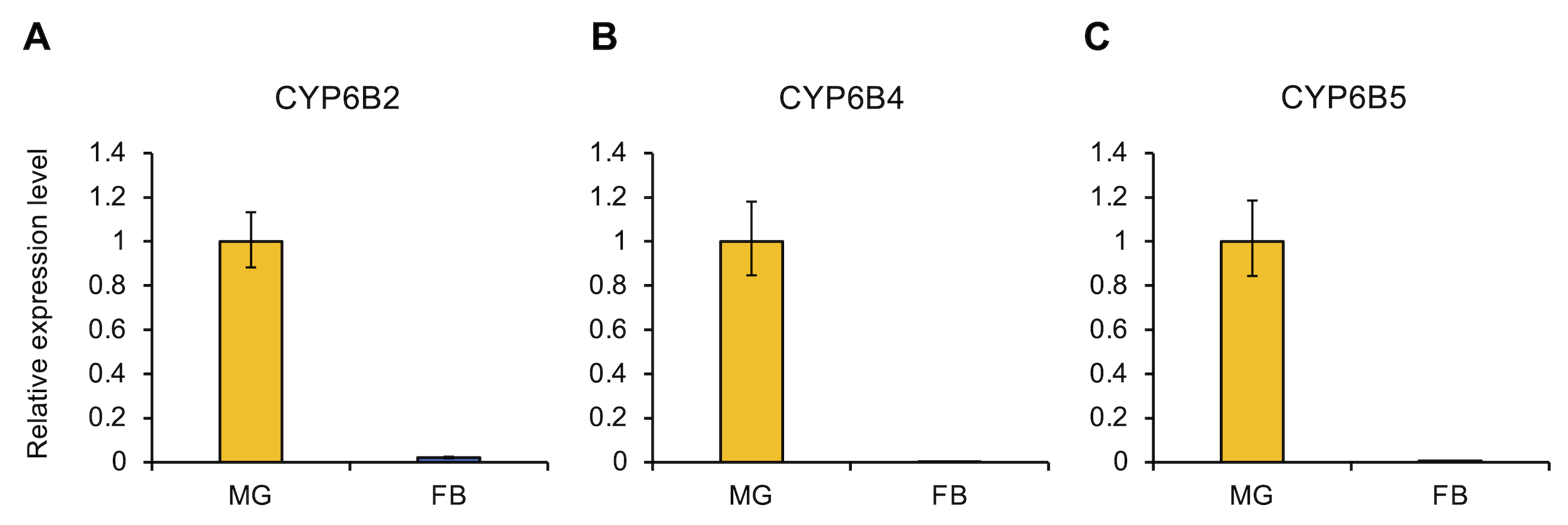

Supplement: S4 Fig — (A-C) The expression levels of CYP6B2 (A), CYP6B4 (B), and CYP6B5 (C) were analyzed by RT–qPCR (MG; midgut, FB; fat bodies). Relative expression levels mean relative quantification (RQ). Bars show RQ minimum and RQ maximum. (TIFF) [file pone.0321438.s004.tiff]

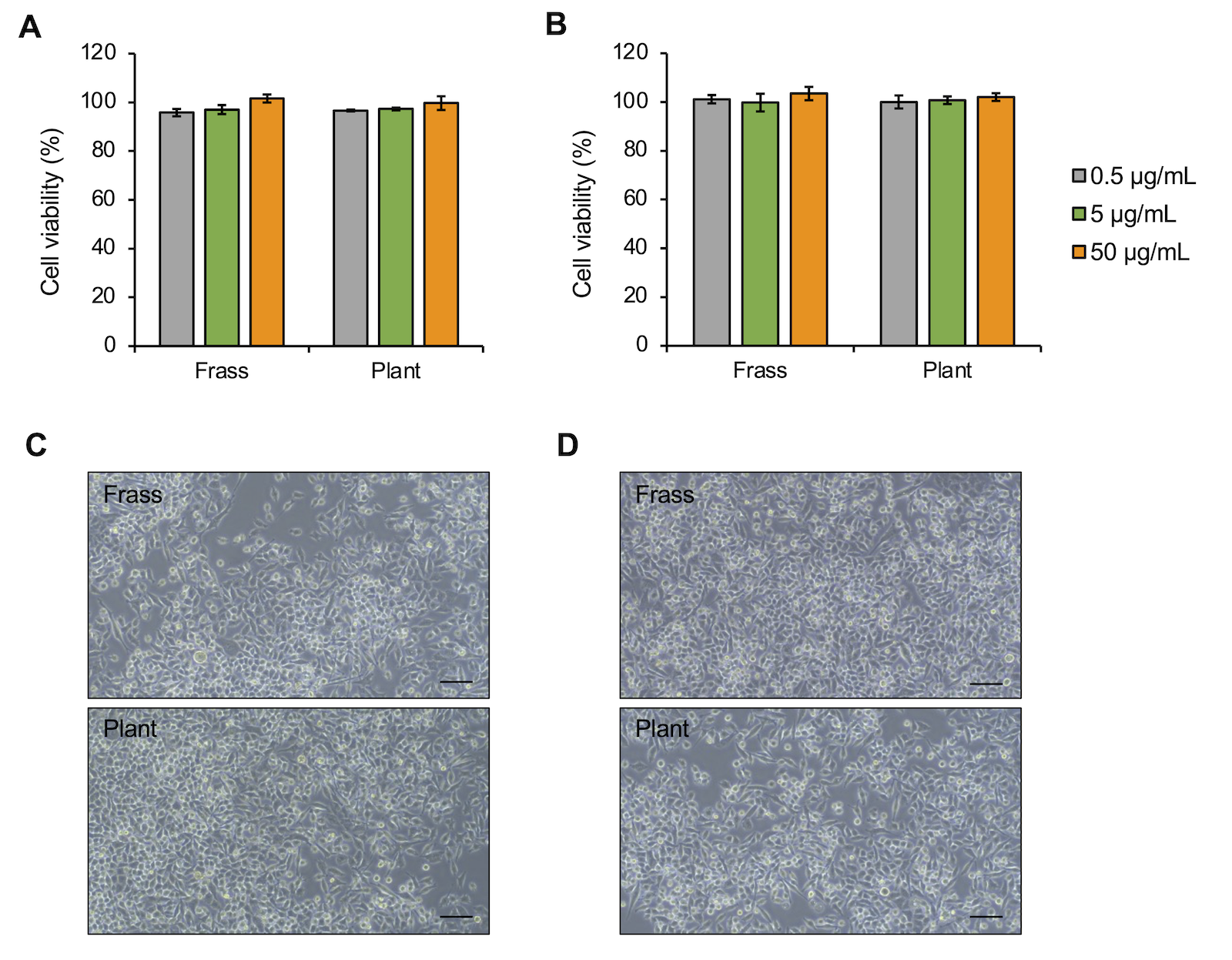

Supplement: S5 Fig — (A-B) Viability of MIA PaCa2 cells relative to that of the control treatment with 0.1% (v/v) DMSO after treatment with Hex. extract (A) and the Met. extract (B) from larval frass and C. paradisi leaves (plant), as determined via the WST-8 assay. The error bars represent the means ± SDs from three biological replicates. (C-D) Cell morphological observations after treatment with Hex. extract (C) and the Met. extract (D) from the larval frass and C. paradisi leaves at 50 µg/mL. Scale bars = 100 μm. (TIFF) [file pone.0321438.s005.tiff]

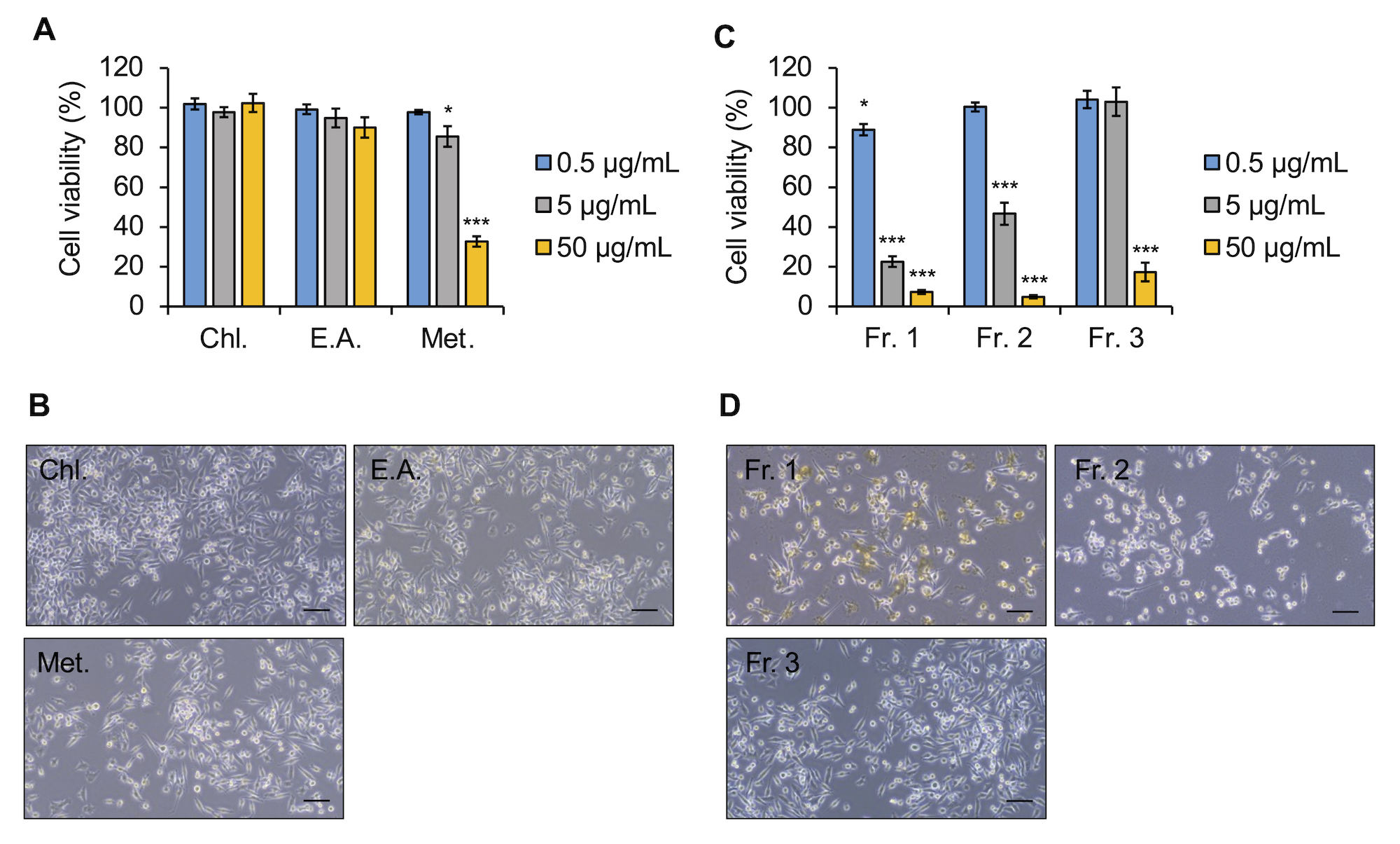

Supplement: S6 Fig — (A) Viability of MIA PaCa2 cells relative to that of the control treatment with 0.2% (v/v) DMSO after treatment with Chl. Fr., E.A. Fr., and Met. Fr. (B) Cell morphological observations after treatment with Chl. Fr., E.A. Fr., and Met. Fr. at 50 µg/mL. (C) Cell viability relative to that of the control treatment with 0.1% (v/v) DMSO after treatment with Fr. 1 to Fr. 3. (D) Cell morphology observation after treatment with Fr. 1 to Fr. 3 at 50 µg/mL. The cell viability was measured via a WST-8 assay. The error bars represent the means ± SDs from three biological replicates. Significant differences from the control were analyzed with Student’s t test, *P < 0.05, ***P < 0.001. Scale bars = 100 μm. (TIFF) [file pone.0321438.s006.tiff]

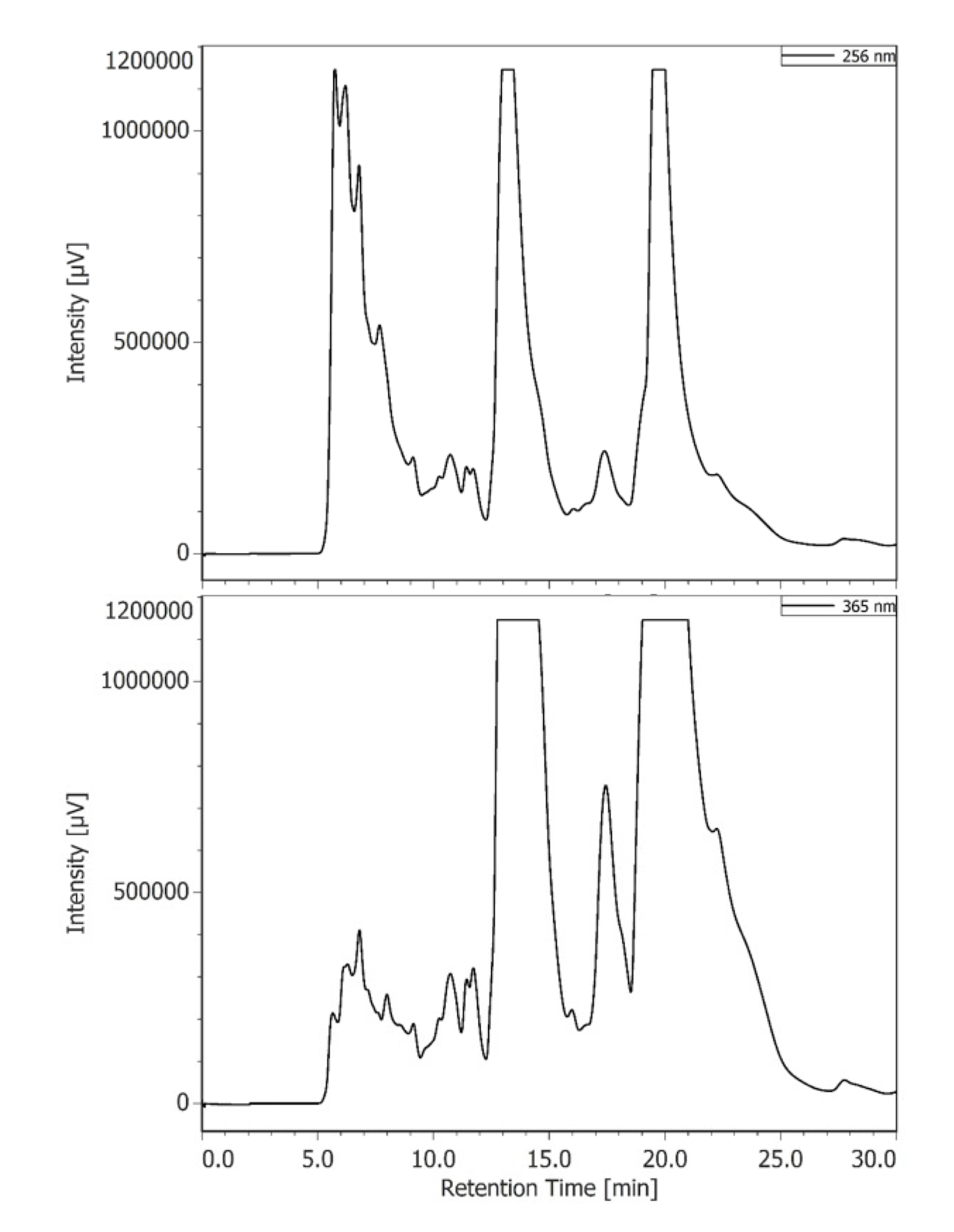

Supplement: S7 Fig — Peaks were detected by 256 nm and 365 nm. (TIFF) [file pone.0321438.s007.tiff]

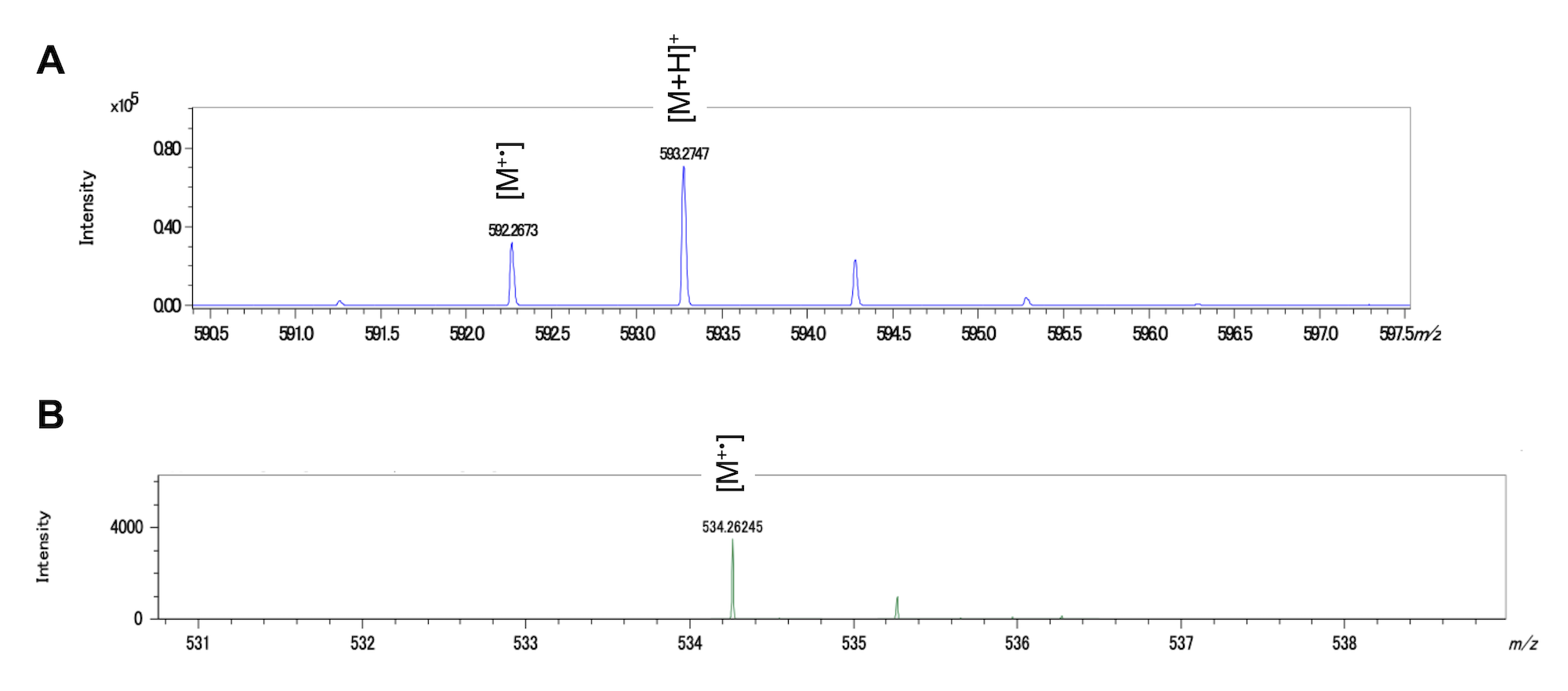

Supplement: S8 Fig — (A) Precursor ion spectrum of Fr. 7 was m/z 592.3. (B) Precursor ion spectrum of Fr. 10 was m/z 534.3. (TIFF) [file pone.0321438.s008.tiff]

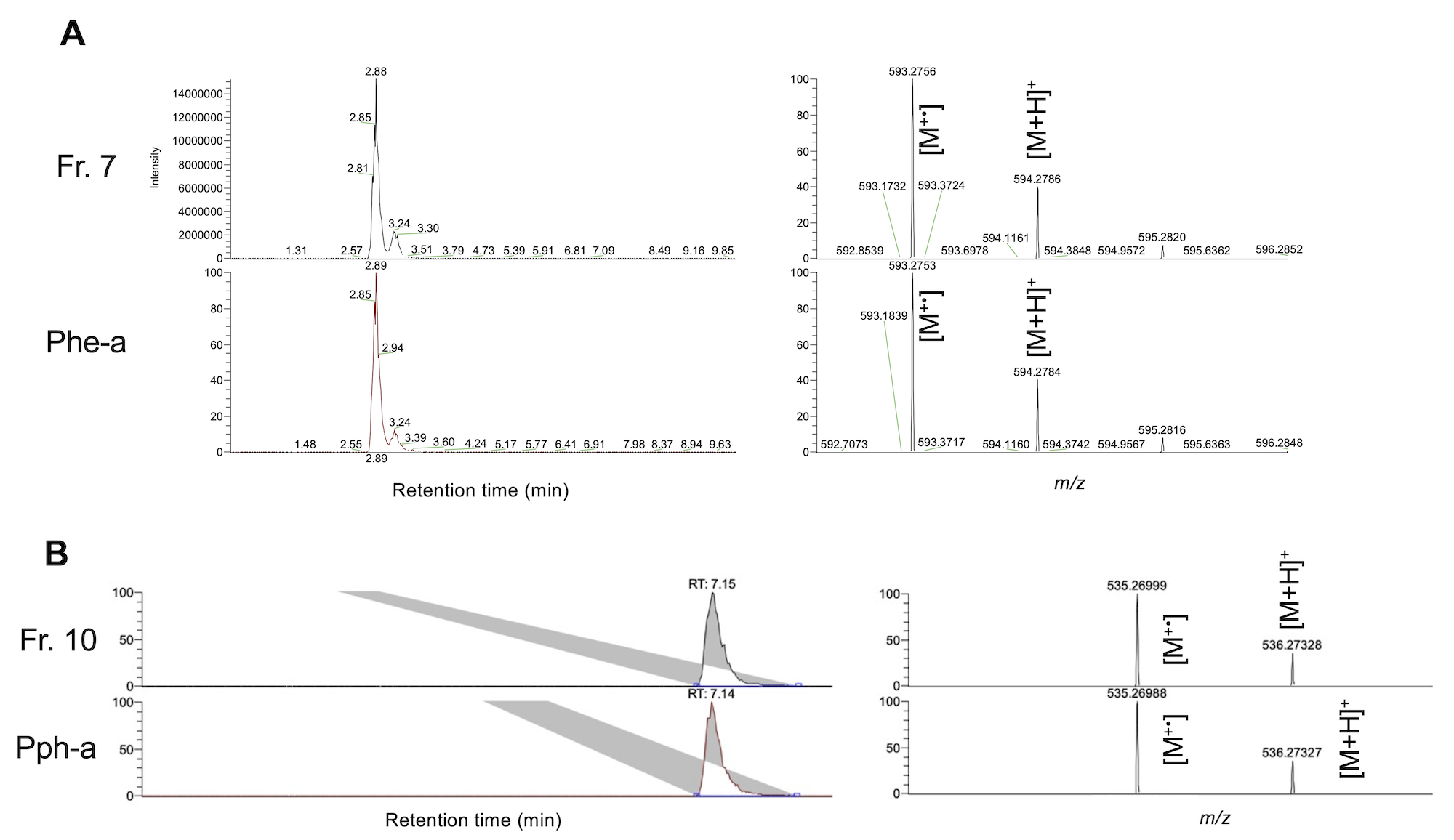

Supplement: S9 Fig — (A) Comparison of Fr. 7 and the Phe-a standard. (B) Comparison of Fr. 10 and the Pph-a standard. Peaks were detected at 366 nm. (TIFF) [file pone.0321438.s009.tiff]
